# QTc interval prolongation by fexofenadine in healthy human volunteers and its correlation with plasma levels of fexofenadine: A demonstration of anticlockwise hysteresis

**DOI:** 10.4103/0253-7613.71919

**Published:** 2010-12

**Authors:** Falgun I. Vyas, Shiv Prakash, A.J. Singh

**Affiliations:** Department of Pharmacology, AMC MET Medical College and Smt. NHL Medical College, Ahmedabad, India

**Keywords:** Drug concentration–effect relationship, fexofenadine, hysteresis, QTc interval prolongation, two-compartment model

## Abstract

**Aim:**

The study was designed to establish relationship between the plasma concentration and QTc interval prolonging effect of fexofenadine and demonstrate the phenomenon of anticlockwise hysteresis.

**Materials and Methods:**

Six subjects were given fexofenadine 60 mg tablet orally under stable conditions, and their drug concentrations were measured at regular intervals. At predetermined time, their ECGs were recorded. Data were analyzed and plotted graphically.

**Design and Setting:**

Randomized parallel design, single group study conducted at clinical research organization of Ahmadabad.

**Results:**

In all subjects time taken for maximum plasma concentration of fexofenadine (T_max_) was around 3 h and the value of average maximum plasma concentration was 460.63 ng/mL, the effect of fexofenadine on the heart (measured as QTc interval prolongation) was maximum (E_max_) after 6 h and average QTc interval was 469.75 ms. Thus, the time to maximum concentration of fexofenadine did not match with the maximum effect on the heart as measured by QTc interval.

**Conclusion:**

The relationship between the drug concentration and drug effect on the heart are at two different time scales. It can be understood by two-compartment model of pharmacokinetics, and this retardation or lagging of an effect behind the concentration is known as hysteresis. The increase of QTc was not beyond 500 ms and not sustained, demonstrating overall cardiac safety of fexofenadine.

## Introduction

Significant cardiac toxicity has been associated with some older antihistamines (e.g., terfenadine and astemizole) when their plasma concentrations are increased, due to which some of them were withdrawn from the market.[1] There is, thus, a need for a thorough assessment of the cardiac safety of newer antihistaminic compounds. Fexofenadine hydrochloride is the synthetic hydrochloride salt of fexofenadine, the carboxylic acid metabolite of terfenadine. It is a second-generation piperidine. It is an orally active non-sedating H1-receptor antagonist. Fexofenadine is the major metabolite of terfenadine in man and is largely responsible for the antihistaminic effect following the administration of terfenadine. Fexofenadine is effective for the relief of symptoms associated with seasonal allergic rhinitis (sneezing, rhinorrhea, pruritus, and lacrimation).[2,3] In clinical studies, this nonsedating antihistamine has been found to relieve symptoms associated with allergic conditions such as seasonal allergic rhinitis.[4,5]

Results of clinical safety and efficacy trials with fexofenadine HCl doses up to 240 mg twice daily in patients with seasonal rhinitis have further demonstrated its safety, indicating a large therapeutic window for this drug.[6] The drug effect is seen within 1 h, achieving maximum effect at 6 h, and lasting a minimum of 12 h after medication,[7,8] and the drug can be used for long periods without evidence of intolerance.[9]

This study was undertaken to assess the relationship of plasma concentration of fexofenadine with the QT interval prolongation.

The QT interval represents the duration of ventricular depolarization and subsequent repolarization, beginning at the initiation of the QRS complex and ending where the T-wave returns to isoelectric baseline.[9] While the degree of QT prolongation is recognized as an imperfect biomarker for proarrhythmic risk, there is a qualitative relationship between QT prolongation and the risk of Torsades De Pointes, especially for drugs that cause substantial prolongation of the QT/QTc interval.[2]

## Materials and Methods

This study was conducted in the Pharmacological Department of Synchron Research, a clinical research organization of Ahmedabad. Ethics Committee approval was obtained for this study by an Institutional Ethics Committee. During the course of a routine bioequivalence study, there were six subjects, who consented to the additional measurement of QT intervals by periodic ECG.

These subjects were between the ages of 21–40 years with weight of >50 kg [but within the range of the LIC (Life Insurance Corporation of India) height–weight chart], heart rate 60–90 beats/min; normal body temperature, respiratory rate (RR) 15–25 breaths/min, systolic blood pressure (SBP) 100–130 mmHg, and diastolic blood pressure (DBP) 50–90 mmHg.

Informed consent was obtained as per ICH–GCP guidelines. The subjects tested negative for drugs of abuse and had routine laboratory investigation values within normal range. The subjects were housed in the department on the previous day of the study, till end of study on the next day. They were free from any renal, hepatic, or cardiac diseases.

The subjects were relaxed and provided standard food, but were asked to abstain from caffeine, tobacco, and alcohol at least 24 h before the vital parameters were recorded (BP, HR, RR, and temperature). This was followed by ECG recording. Consistent operator technique was maintained by ensuring skin preparation, correct leads placement, subject position, and data acquisition practice.

ECG was recorded with validated electronic device, “NASAN-ECG1997” with paper speed 25 mm/s, calibration 10 mm/mv.

The ECGs were recorded by an inbuilt software programme, which also calculates QTc by Bazett’s formula (QT/square root of R–R) where QTc is the rate corrected in milliseconds and R–R interval in seconds.[10]

Blood (9 mL) was sampled from antecubital or cubital veins and collected into sodium citrate-containing tubes at 0, 1, 2, 3, 4, 6, 8, and 10 h after the administration of each fexofenadine tablet formulation (180 mg). The blood samples were centrifuged at 2500g for 10 min at 4°C, and the separated plasma was collected and stored at −20°C until drug analysis.

All plasma samples were analyzed for fexofenadine concentration according to a sensitive, selective, and accurate high-performance liquid chromatography (HLPC) method, which was developed and validated before the study. The chromatographic separations and quantitative determination were performed using a high-performance liquid chromatograph from E. Merck, equipped with a degassing unit, quaternary pump, autoinjector, UV detector, and controlled by Merck software.

Chromatographic separation was performed using a C18 (150 × 4.6 mm^2^id.; 5 μm particle size) HPLC column. The mobile phase consisted of acetonitrile and potassium dihydrogen phosphate (0.05 mol/l, pH 5.0). The mobile phase was eluted at a flow rate of 1.4 mL/min at 52°C. UV detection of fexofenadine and the internal standard (cetirizine) was performed at 195 nm. Each analysis required a maximum of 13 min. The calibration curves were linear over a range of 10–1000 ng/mL using 1.0 mL plasma samples. All samples from each volunteer were measured on the same day in order to avoid inter-assay variation.

The ECG’s were also performed at just after collection of blood samples. QTc intervals were read off from the automated ECG’s obtained. The values of plasma concentrations and QTc intervals were recorded [Table 1]; and mean data compared [Table 2].

**Table 1:** Plasma concentrations and QTc interval in individual patients

*Time/h*	*Patient 1*		*Patient 2*		*Patient 3*		*Patient 4*		*Patient 5*		*Patient 6*	
	*Conc. in blood in ng/ mL*	*QTc in ms*	*Conc. in blood in ng/ mL*	*QTc in ms*	*Conc. in blood in ng/ mL*	*QTc in ms*	*Conc. in blood in ng/ mL*	*QTc in ms*	*Conc. in blood in ng/ mL*	*QTc in ms*	*Conc. in blood in ng/ mL*	*QTc in ms*
0	NA	430	NA	444	NA	426	NA	457	NA	405	NA	415
1	240.36	410	207.53	439	158.70	421	254.12	454	85.23	409	161.83	407
2	346.87	430	388.64	429	411.89	408	436.84	436	250.02	414	334.38	415
3	483.52	410	498.21	424	422.56	398	510.56	439	425.87	399	422.83	398
4	436.20	441	443.62	429	458.15	392	410.89	434	392.56	402	405.36	418
6	400	450	372.11	490	380.56	412	322.87	537	295.02	480	326.43	448
8	264.25	438	283.92	455	310.25	439	290.56	441	210.36	436	253.06	421
10	212.02	443	222.84	451	195	442	236.54	452	195.21	432	196.31	433

**Table 2:** Comparison of mean drug concentrations and QTc intervals

*Time/h*	*Drug concentration in ng/mL as mean ± SD*	*QTc interval in milliseconds as mean ± SD*
0	0	429.50 ± 18.91
1	184.63 ± 62.50	423.33 ± 19.19
2	361.44 ± 66.83	422 ± 11.12
3	460.59 ± 41.26	411.33 ± 16.94
4	424.46 ± 25.32	419.33 ± 19.12
6	349.50 ± 40.58	469.50 ± 42.98
8	268.73 ± 34.94	438.33 ± 10.88
10	209.65 ± 17.34	442.17 ± 8.52

**Figure 1:**
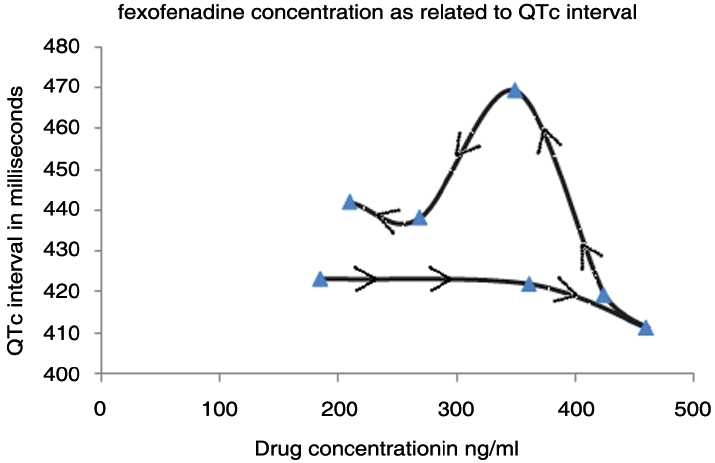
Showing anticlockwise hysteresis, triangles show relationship between drug concentration and QTc interval while the direction of arrow shows relationship between two with progress of time which is anticlockwise direction.

The data were graphically plotted, with QTc intervals on the ordinate and drug concentration on the abscissa. The resulting graph is depicted [Figure 1]. The arrow suggests the change of drug concentration with time in anticlockwise direction so this is anticlockwise hysteresis.

## Results

The plot of drug concentration in ng/mL and QTc interval in milliseconds demonstrated counter-clockwise hysteresis for fexofenadine.

Fexofenadine did not prolong QTc in dogs when administered at a dosage of 10 mg/kg/day orally for 5 days or in rabbits when administered at a dosage producing plasma concentrations at least 28 and 63 times greater than those seen after administration of therapeutic dosages (60 mg twice daily) in humans.[3,11]

This lack of propensity to cause QTc irregularities has been confirmed in two long-term studies in volunteers. Fexofenadine 60 mg twice daily for 6 months or 240 mg once daily for 12 months had no significant effect on QTc relative to placebo.[11,12]

The maximum concentration of fexofenadine (*C*_max_) reaches in 3 h after oral administration of fexofenadine tablets, and the maximum effect on heart (*E*_max_) as QTc interval prolongation takes 6 h after oral administration of the drug, so the cause of this time lag can be explained by a two-compartment model.[13,14]

When this data were analyzed by paired *t*-test, comparing mean of drug concentration in plasma and mean of QTc interval; *P* value was found to be 0.028 (<0.05). Thus, a statistically significant relationship existed between these two parameters.

Fexofenadine has high volume of distribution; *V*_d_(5.4–5.8 L/kg).[15] In the case of drugs with slow distribution phase, initially the plasma concentrations are high for few hours; after which they fall as the drug redistributes and then falls more slowly due to elimination. The site of action of fexofenadine is in a compartment to which drug is distributed slowly. It may be recalled that severe ventricular arrhythmias, i.e., Torsades de pointes developed 15 h after ingestion of terfenadine. Therefore, cardiac monitoring for at least 24 h is recommended along with standard measures to remove any unabsorbed drug.[16]

Although fexofenadine is relatively safer, the clinical significance of the hysteresis phenomenon seen in the case of fexofenadine is that the monitoring of drug concentration level in plasma will not be helpful in deciding its effects and ECG monitoring for such drugs affecting QTc interval should be done ideally between 4 and 8 h after dosing in individuals at risk.

## Discussion

Hysteresis may be defined as “the retardation or lagging of an effect behind the cause of the effect.” The two main reasons for the lag phase are limited access to the site of drug action or slow receptor kinetics. Both these characteristics produce an anticlockwise hysteresis, in which time moves anticlockwise in the change in the relationship between plasma concentration and observed effect with time.[16] A clockwise hysteresis occurs on the other hand, as in drug tolerance. In delayed distribution, the site of drug action is at a site to which the drug is slowly distributed. This effect increases as the drug concentration falls due to redistribution to tissues in blood. Drug concentrations soon after a dose cause a smaller effect than the same concentrations later when distribution to the site of action has occurred. This results in an anticlockwise hysteresis in the concentration–effect relationship.

The values of *E*_max_(6 h) is in agreement with related studies in literature.[17] The results in our study plotted graphically show an anticlockwise hysteresis loop, which could indicate equilibration delay between the plasma concentration and effect site, requiring an effect site compartment in the pharmacodynamic model; the high volume of distribution of fexofenadine (5.4–5.8 L/kg) confirms this.
